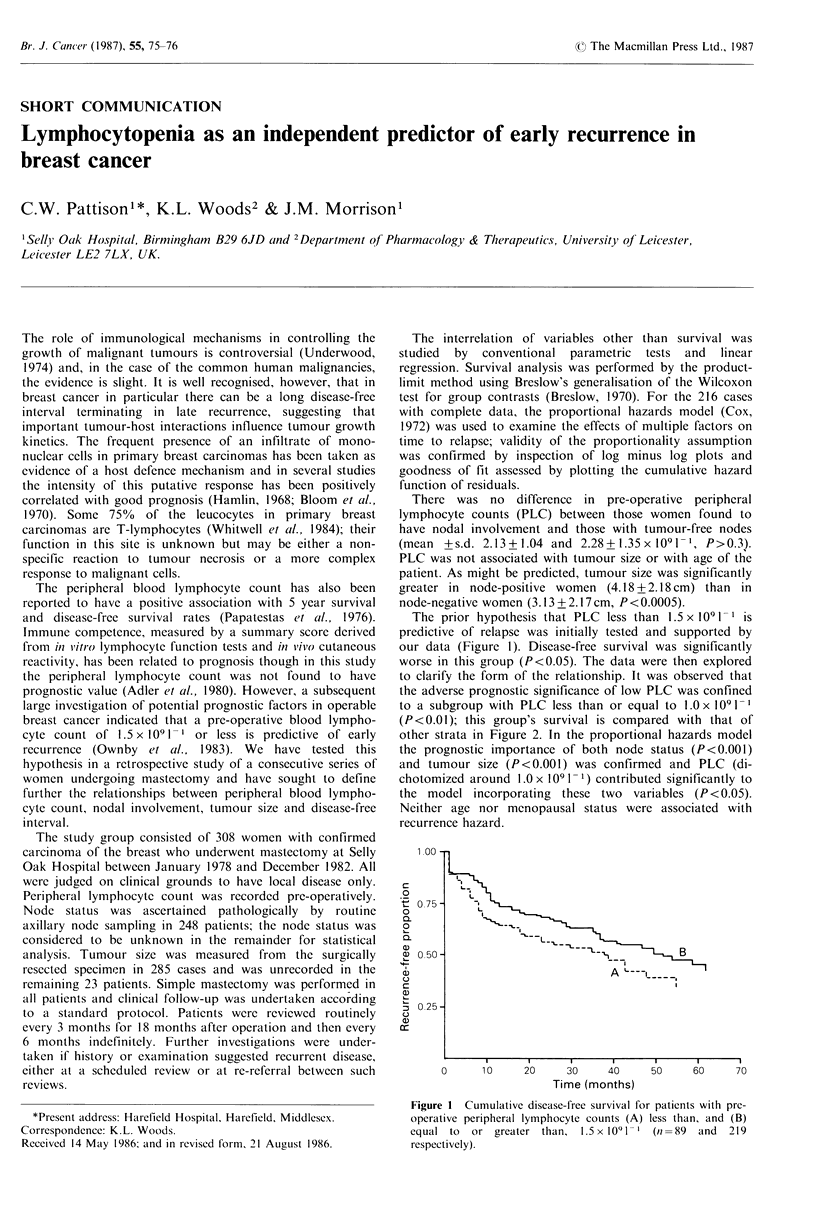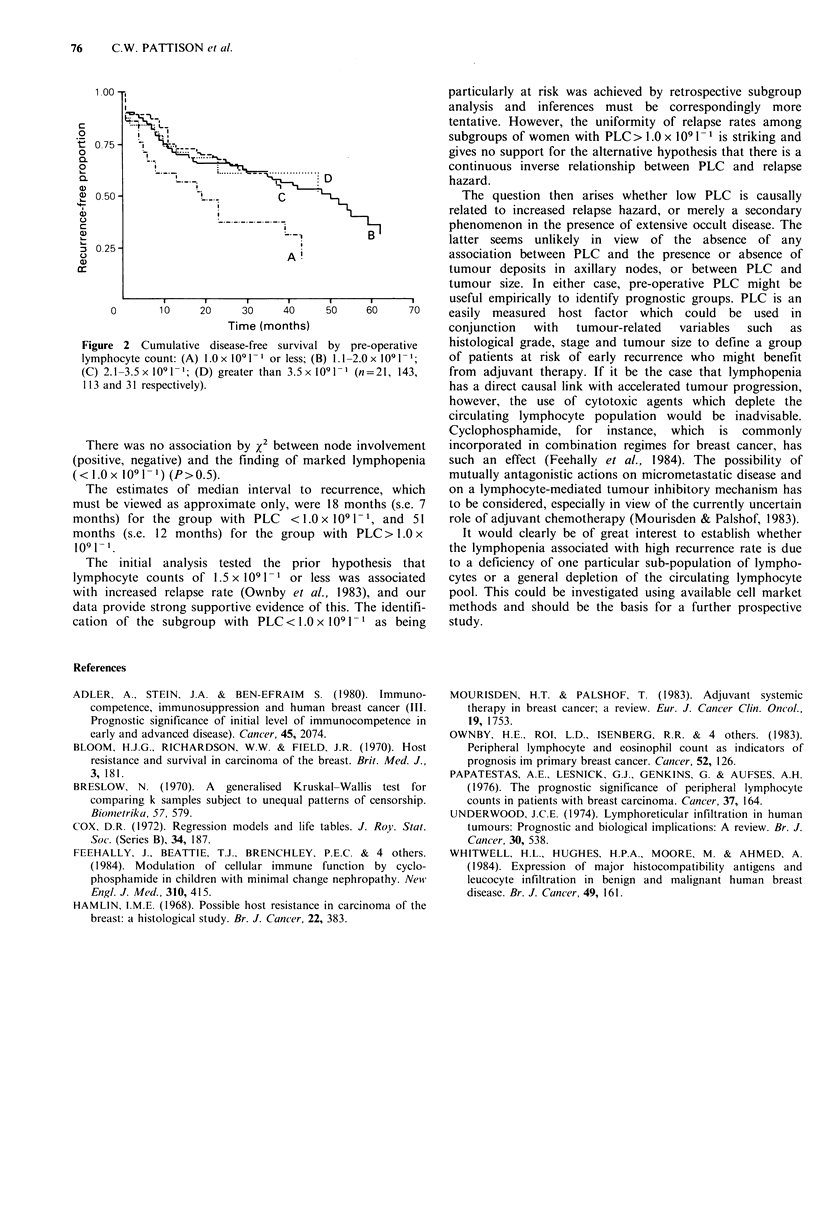# Lymphocytopenia as an independent predictor of early recurrence in breast cancer.

**DOI:** 10.1038/bjc.1987.15

**Published:** 1987-01

**Authors:** C. W. Pattison, K. L. Woods, J. M. Morrison


Br. J. Cancer (1987), 55, 75–76

© The Macmillan Press Ltd., 1987

SHORT COMMUNICATION

### Lymphocytopenia as an independent predictor of early recurrence in breast cancer

C.W. Pattison[1]*, K.L. Woods[2] & J.M. Morrison[1]

[1]*Selly Oak Hospital, Birmingham B29 6JD and* [2]*Department of Pharmacology & Therapeutics, University of Leicester, Leicester LE2 7LX, UK.*

The role of immunological mechanisms in controlling the growth of malignant tumours is controversial (Underwood, 1974) and, in the case of the common human malignancies, the evidence is slight. It is well recognised, however, that in breast cancer in particular there can be a long disease-free interval terminating in late recurrence, suggesting that important tumour-host interactions influence tumour growth kinetics. The frequent presence of an infiltrate of mononuclear cells in primary breast carcinomas has been taken as evidence of a host defence mechanism and in several studies the intensity of this putative response has been positively correlated with good prognosis (Hamlin, 1968; Bloom *et al.*, 1970). Some 75% of the leucocytes in primary breast carcinomas are T-lymphocytes (Whitwell *et al.*, 1984); their function in this site is unknown but may be either a non-specific reaction to tumour necrosis or a more complex response to malignant cells.

The peripheral blood lymphocyte count has also been reported to have a positive association with 5 year survival and disease-free survival rates (Papatestas *et al.*, 1976). Immune competence, measured by a summary score derived from *in vitro* lymphocyte function tests and *in vivo* cutaneous reactivity, has been related to prognosis though in this study the peripheral lymphocyte count was not found to have prognostic value (Adler *et al.*, 1980). However, a subsequent large investigation of potential prognostic factors in operable breast cancer indicated that a pre-operative blood lymphocyte count of $1.5 \times 10^9 l^{-1}$ or less is predictive of early recurrence (Ownby *et al.*, 1983). We have tested this hypothesis in a retrospective study of a consecutive series of women undergoing mastectomy and have sought to define further the relationships between peripheral blood lymphocyte count, nodal involvement, tumour size and disease-free interval.

The study group consisted of 308 women with confirmed carcinoma of the breast who underwent mastectomy at Selly Oak Hospital between January 1978 and December 1982. All were judged on clinical grounds to have local disease only. Peripheral lymphocyte count was recorded pre-operatively. Node status was ascertained pathologically by routine axillary node sampling in 248 patients; the node status was considered to be unknown in the remainder for statistical analysis. Tumour size was measured from the surgically resected specimen in 285 cases and was unrecorded in the remaining 23 patients. Simple mastectomy was performed in all patients and clinical follow-up was undertaken according to a standard protocol. Patients were reviewed routinely every 3 months for 18 months after operation and then every 6 months indefinitely. Further investigations were undertaken if history or examination suggested recurrent disease, either at a scheduled review or at re-referral between such reviews.

*Present address: Harefield Hospital, Harefield, Middlesex.
Correspondence: K.L. Woods.

Received 14 May 1986; and in revised form, 21 August 1986.

The interrelation of variables other than survival was studied by conventional parametric tests and linear regression. Survival analysis was performed by the product-limit method using Breslow's generalisation of the Wilcoxon test for group contrasts (Breslow, 1970). For the 216 cases with complete data, the proportional hazards model (Cox, 1972) was used to examine the effects of multiple factors on time to relapse; validity of the proportionality assumption was confirmed by inspection of log minus log plots and goodness of fit assessed by plotting the cumulative hazard function of residuals.

There was no difference in pre-operative peripheral lymphocyte counts (PLC) between those women found to have nodal involvement and those with tumour-free nodes (mean $\pm$s.d. $2.13 \pm 1.04$ and $2.28 \pm 1.35 \times 10^9 l^{-1}$, $P > 0.3$). PLC was not associated with tumour size or with age of the patient. As might be predicted, tumour size was significantly greater in node-positive women ($4.18 \pm 2.18$ cm) than in node-negative women ($3.13 \pm 2.17$ cm, $P < 0.0005$).

The prior hypothesis that PLC less than $1.5 \times 10^9 l^{-1}$ is predictive of relapse was initially tested and supported by our data (Figure 1). Disease-free survival was significantly worse in this group ($P < 0.05$). The data were then explored to clarify the form of the relationship. It was observed that the adverse prognostic significance of low PLC was confined to a subgroup with PLC less than or equal to $1.0 \times 10^9 l^{-1}$ ($P < 0.01$); this group's survival is compared with that of other strata in Figure 2. In the proportional hazards model the prognostic importance of both node status ($P < 0.001$) and tumour size ($P < 0.001$) was confirmed and PLC (dichotomized around $1.0 \times 10^9 l^{-1}$) contributed significantly to the model incorporating these two variables ($P < 0.05$). Neither age nor menopausal status were associated with recurrence hazard.

**Figure 1** Cumulative disease-free survival for patients with pre-operative peripheral lymphocyte counts (A) less than, and (B) equal to or greater than, $1.5 \times 10^9 l^{-1}$ ($n = 89$ and 219 respectively).

**Figure 2** Cumulative disease-free survival by pre-operative lymphocyte count: (A) $1.0 \times 10^9 l^{-1}$ or less; (B) $1.1–2.0 \times 10^9 l^{-1}$; (C) $2.1–3.5 \times 10^9 l^{-1}$; (D) greater than $3.5 \times 10^9 l^{-1}$ ($n=21$, 143, 113 and 31 respectively).

There was no association by $\chi^2$ between node involvement (positive, negative) and the finding of marked lymphopenia ($<1.0 \times 10^9 l^{-1}$) ($P>0.5$).

The estimates of median interval to recurrence, which must be viewed as approximate only, were 18 months (s.e. 7 months) for the group with PLC $<1.0 \times 10^9 l^{-1}$, and 51 months (s.e. 12 months) for the group with PLC$>1.0 \times 10^9 l^{-1}$.

The initial analysis tested the prior hypothesis that lymphocyte counts of $1.5 \times 10^9 l^{-1}$ or less was associated with increased relapse rate (Ownby *et al.*, 1983), and our data provide strong supportive evidence of this. The identification of the subgroup with PLC$<1.0 \times 10^9 l^{-1}$ as being particularly at risk was achieved by retrospective subgroup analysis and inferences must be correspondingly more tentative. However, the uniformity of relapse rates among subgroups of women with PLC$>1.0 \times 10^9 l^{-1}$ is striking and gives no support for the alternative hypothesis that there is a continuous inverse relationship between PLC and relapse hazard.

The question then arises whether low PLC is causally related to increased relapse hazard, or merely a secondary phenomenon in the presence of extensive occult disease. The latter seems unlikely in view of the absence of any association between PLC and the presence or absence of tumour deposits in axillary nodes, or between PLC and tumour size. In either case, pre-operative PLC might be useful empirically to identify prognostic groups. PLC is an easily measured host factor which could be used in conjunction with tumour-related variables such as histological grade, stage and tumour size to define a group of patients at risk of early recurrence who might benefit from adjuvant therapy. If it be the case that lymphopenia has a direct causal link with accelerated tumour progression, however, the use of cytotoxic agents which deplete the circulating lymphocyte population would be inadvisable. Cyclophosphamide, for instance, which is commonly incorporated in combination regimes for breast cancer, has such an effect (Feehally *et al.*, 1984). The possibility of mutually antagonistic actions on micrometastatic disease and on a lymphocyte-mediated tumour inhibitory mechanism has to be considered, especially in view of the currently uncertain role of adjuvant chemotherapy (Mourisden & Palshof, 1983).

It would clearly be of great interest to establish whether the lymphopenia associated with high recurrence rate is due to a deficiency of one particular sub-population of lymphocytes or a general depletion of the circulating lymphocyte pool. This could be investigated using available cell market methods and should be the basis for a further prospective study.

### References

ADLER, A., STEIN, J.A. & BEN-EFRAIM S. (1980). Immunocompetence, immunosuppression and human breast cancer (III. Prognostic significance of initial level of immunocompetence in early and advanced disease). *Cancer*, **45**, 2074.

BLOOM, H.J.G., RICHARDSON, W.W. & FIELD, J.R. (1970). Host resistance and survival in carcinoma of the breast. *Brit. Med. J.*, **3**, 181.

BRESLOW, N. (1970). A generalised Kruskal–Wallis test for comparing k samples subject to unequal patterns of censorship. *Biometrika, 57*, 579.

COX, D.R. (1972). Regression models and life tables. *J. Roy. Stat. Soc.* (Series B), **34**, 187.

FEEHALLY, J., BEATTIE, T.J., BRENCHLEY, P.E.C. & 4 others. (1984). Modulation of cellular immune function by cyclophosphamide in children with minimal change nephropathy. *New Engl. J. Med.*, **310**, 415.

HAMLIN, I.M.E. (1968). Possible host resistance in carcinoma of the breast: a histological study. *Br. J. Cancer*, **22**, 383.

MOURISDEN, H.T. & PALSHOF, T. (1983). Adjuvant systemic therapy in breast cancer; a review. *Eur. J. Cancer Clin. Oncol.*, **19**, 1753.

OWNBY, H.E., ROI, L.D., ISENBERG, R.R. & 4 others. (1983). Peripheral lymphocyte and eosinophil count as indicators of prognosis im primary breast cancer. *Cancer*, **52**, 126.

PAPATESTAS, A.E., LESNICK, G.J., GENKINS, G. & AUFSES, A.H. (1976). The prognostic significance of peripheral lymphocyte counts in patients with breast carcinoma. *Cancer*, **37**, 164.

UNDERWOOD, J.C.E. (1974). Lymphoreticular infiltration in human tumours: Prognostic and biological implications: A review. *Br. J. Cancer*, **30**, 538.

WHITWELL, H.L., HUGHES, H.P.A., MOORE, M. & AHMED, A. (1984). Expression of major histocompatibility antigens and leucocyte infiltration in benign and malignant human breast disease. *Br. J. Cancer*, **49**, 161.